# Epithelial to Mesenchymal Transition in Patients with Pancreatic Ductal Adenocarcinoma: State-of-the-Art and Therapeutic Opportunities

**DOI:** 10.3390/ph14080740

**Published:** 2021-07-29

**Authors:** Julie Dardare, Andréa Witz, Jean-Louis Merlin, Agathe Bochnakian, Paul Toussaint, Pauline Gilson, Alexandre Harlé

**Affiliations:** Université de Lorraine, CNRS UMR7039 CRAN, Service de Biopathologie, Institut de Cancérologie de Lorraine, 54519 Vandoeuvre-lès-Nancy, France; a.witz@nancy.unicancer.fr (A.W.); jl.merlin@nancy.unicancer.fr (J.-L.M.); a.bochnakian@nancy.unicancer.fr (A.B.); p.toussaint@nancy.unicancer.fr (P.T.); p.gilson@nancy.unicancer.fr (P.G.); a.harle@nancy.unicancer.fr (A.H.)

**Keywords:** PDAC, EMT, metastasis, biomarker

## Abstract

Pancreatic ductal adenocarcinoma (PDAC) is one of the malignancies with the worst prognosis despite a decade of efforts. Up to eighty percent of patients are managed at late stages with metastatic disease, in part due to a lack of diagnosis. The effectiveness of PDAC therapies is challenged by the early and widespread metastasis. Epithelial to mesenchymal transition (EMT) is a major driver of cancer progression and metastasis. This process allows cancer cells to gain invasive properties by switching their phenotype from epithelial to mesenchymal. The importance of EMT has been largely described in PDAC, and its importance is notably highlighted by the two major subtypes found in PDAC: the classical epithelial and the quasi-mesenchymal subtypes. Quasi-mesenchymal subtypes have been associated with a poorer prognosis. EMT has also been associated with resistance to treatments such as chemotherapy and immunotherapy. EMT is associated with several key molecular markers both epithelial and mesenchymal. Those markers might be helpful as a biomarker in PDAC diagnosis. EMT might becoming a key new target of interest for the treatment PDAC. In this review, we describe the role of EMT in PDAC, its contribution in diagnosis, in the orientation and treatment follow-up. We also discuss the putative role of EMT as a new therapeutic target in the management of PDAC.

## 1. Introduction

Pancreatic cancer is the fourth-leading cause of death by cancer, and has the lowest 5-year relative survival rate (9%) as reported by The American Cancer Society reports in 2020 [1]. The incidence of pancreatic cancer continue to increase, and it is projected to become the second cause of cancer death before 2030 in Western countries [2]. The pancreatic ductal adenocarcinoma (PDAC) histological subtype represents almost 90% of pancreatic malignancies. Because of the lack of clinical symptoms in early stages and an high metastatic potential of PDAC cells, up to 80% patients are diagnosed at late stages [3]. The majority of the 15–20% patients eligible for a surgical resection finally relapse or develop a local or metastatic recurrence [3,4]. There is an urgent need to improve diagnosis with efficient prognostic biomarkers which would allow a better management of this disease.

Metastatic evolution remains a concern for the management of patients with PDAC. Epithelial to mesenchymal transition (EMT) is one of the key mechanisms that leads to tumor progression and development of metastasis [5,6]. EMT is a dynamic process in which epithelial cells loss their phenotype and acquire a mesenchymal phenotype. This transition is defined by loss of characteristic epithelial markers such as E-cadherin or cytokeratins and a gain of mesenchymal markers such as N-cadherin or vimentin [7]. These changes provide morphological modifications with remodeling of the cytoskeleton, disruption of cell adhesion capacity to other cells, and to the matrix, loss of cellular polarity. Taken together these events enhance invasiveness, migration and finally metastasis [5,6,8].

Two different EMT states have been described in PDAC in vivo models and define the cell dissemination type: partial EMT (pEMT) and complete EMT (cEMT) [9]. In pEMT, cells are stably or dynamically in an epithelial–mesenchymal intermediate state. Cells can express both epithelial and mesenchymal markers or they can loss epithelial features without a gain of mesenchymal features.

Furthermore, EMT has been identified as having a role at preneoplastic stage of PDAC in vivo, allowing cells to seeding to distant organs prior or in parallel to primary tumor formation [10]. Almost all patients with complete surgical resection and no metastasis finally die from disease within five years is consistent with this early spread model [3,11,12]

Taken together this information supports a strong role of EMT in pancreatic cancer progression, contributing to the poor prognosis. Herein, we discuss EMT and its role in PDAC, its interest as a biomarker, or as a therapeutic target.

## 2. Epithelial to Mesenchymal Transition

EMT is a dynamic biological process in which epithelial cells evolves to a mesenchymal state. Epithelial cells normally interact with basement membrane through a basal-apical polarity, they are in close contact with each other through cell–cell junctions. During EMT, epithelial cells undergo changes in gene expression through multiple molecular processes leading to the repression of these epithelial characteristics and gain of mesenchymal features allowing cells migrative and invasive properties [13]. EMT is considered as a dynamic process because of its capacity to reverse the phenomenon through the mesenchymal–epithelial transition (MET), which is less understood. EMT plays crucial roles in the development and evolution of the disease: Three subtypes have been proposed: during implantation, embryogenesis, and organ development (type 1); during tissue regeneration and fibrosis (type 2); and associated with cancer progression and metastasis (type 3) [7].

During cancer progression, type 3 EMT allows carcinoma cells capacities to dissociate themselves from the primary tumor. Then, cells can disseminate through invasion, intravasation, survival in blood and lymphatic stream, extravasation, to finally develop a metastasis into secondary organs. Once at the metastatic site, cells that acquired mesenchymal-like phenotype through EMT seem to reverse the process (MET) to regain epithelial properties and to integrate into distant organs. This phenomenon can be illustrated by the fact that distant metastases are commonly composed of differentiated epithelial cells, however the implication of MET is still being debated [14].

Besides cellular migration, EMT plays a role in a myriad of processes implied in cancer pathways such as resistance to cell death, blocking senescence, enhancing survival, promoting genomic instability, metabolism modifications, drug resistance, and immune suppression [15]. Implication of EMT in cancer progression and metastasis appears to be different according to cancer type.

A new class of drugs termed “migrastatics” has been defined recently: These drugs interfere with all modes of cancer cell invasion and with their ability to metastasize [16]. Unlike conventional cancer drugs which target the proliferation of cancer cells, migrastatics focus on the inhibition of local invasion and metastasis. Recent work has developed a pipeline approach suitable for the development of migrastatics drugs in melanoma [17]. This theoretical approach might comprise EMT and might represent a promising new therapeutical strategy in cancer with high metastatic potential including PDAC.

### EMT Signaling

**Figure 1:**
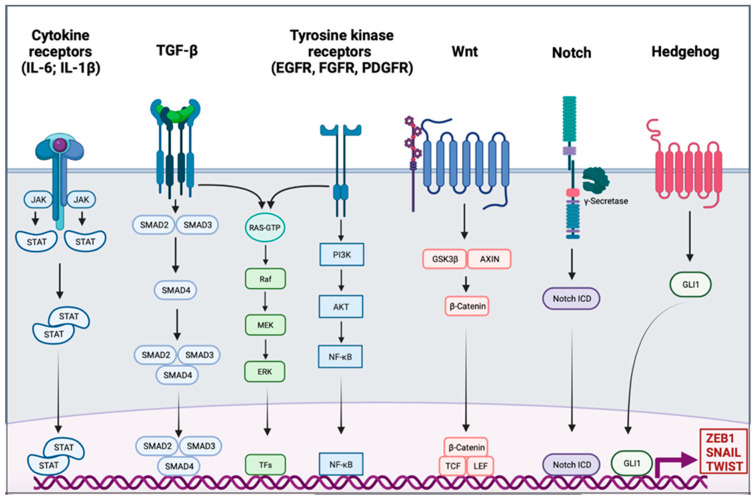
Signaling pathway involved in the epithelial to mesenchymal transition. Different signaling pathways can activate epithelial to mesenchymal transition (EMT) through the activation of EMT transcription factors ZEB1, SNAIL, and TWIST. Interleukin 6 (IL-6) and interleukin 1β (IL-1β) can bind cytokine receptors. Signaling is conducted through the activation of Janus kinase (JAK) and the recruitment of signal transducer and activator of transcription proteins (STATs); the dimer of STATs translocates into the nucleus to activate the transcription of genes. The transforming growth factor β (TGF-β) signal is conducted by SMADs protein into the nucleus, and the trimer activates the transcription. Tyrosine kinase receptors (RTK), such as epidermal growth factor receptor (EGFR), fibroblast growth factor receptor (FGFR), or platelet-derived growth factor receptor (PDGFR), induce PI3K, AKT, and nuclear factor-κB (NF-κB). The TGF-β pathway and RTK are also able to trigger the RAS-RAF-MEK-ERK signaling pathway. The WNT signaling results in the release of β-Catenin from the glycogen synthase kinase-3β (GSK3β)–axis inhibition protein (AXIN) complex. β-Catenin moves into the nucleus and binds to the transcription factors T cell factor (TCF) and the lymphoid enhancer-binding factor (LEF). Intracellular domain of the notch receptor (Notch ICD) is cleaved after the activation of the receptor, then it can translocate into the nucleus and act as a transcriptional co-activator. Hedgehog signaling induces EMT-associated gene expression through the activation of GLI1.

Activation of EMT might be triggered by various signaling pathways depending on the tumor microenvironment. Indeed, tumor-associated stroma can increase the expression of EMT-transcription factors (EMT-TFs). Among signaling pathways are included transforming growth factor β (TGF-β), bone morphogenic protein (BMP), Notch, Wnt/β-catenin, sonic hedgehog, epidermal growth factor (EGF), fibroblast growth factor (FGF), and platelet-derived growth factor (PDGF) [18,19,20,21,22] (Figure 1). Activation of these EMT-inducing signaling pathways leads to the expression of transcription factors that governs EMT-associated genes. They simultaneously repressed the expression of epithelial genes, and on the other hand they induce genes associated with the mesenchymal phenotype. EMT-TFs include basic helix-loop-helix (bHLH) factors TWIST1 and TWIST2, the zinc finger E-box-binding homebox ZEB1 and ZEB2, and the zinc finger binding transcription factors SNAI1 and SNAI2.

SNAIL is the first described transcriptional repressor of E-cadherin; it binds to the E box consensus sequence in the promoter of *CDH1*, encoding E-cadherin, and directly repress its transcription [23,24]. SNAIL also induces the downregulation of others epithelial molecules such as Claudins, Occludins, and Mucin-1. SNAIL also has the ability to directly induce mesenchymal genes such as Fibronectin and Matrix Metallopeptidase 9 (MMP9) [25]. ZEB1 and ZEB2 repress E-cadherin expression by directly binding to the E-Box element of *CDH1* [26]. They also induce the expression of mesenchymal proteins such as N-cadherin and Vimentin [27,28]. Unlike the last two EMT-TFs, TWIST acts as an indirect repressor of E-cadherin partly due to its transcriptional activation of SNAI2 [29,30]. TWIST is also able to activate expression of mesenchymal genes such as N-cadherin and Vimentin [31]. The functional loss of E-cadherin is considered as a crucial step in EMT; however, many others epithelial proteins are also downregulated: Mucin-1, Occludins, Claudins, and Desmoplakin. On the other side, mesenchymal markers are gained, they include N-cadherin, Vimentin, Smooth Muscle Actin, Fibronectin, Matrix Metalloproteinases, and Vitronectin [32]. In addition to its role in transcriptional regulation, EMT might be orchestrated by other regulatory networks including regulation by microRNAs (miRs), differential splicing, translational and posttranslational control.

Molecular changes described above lead to cellular hallmarks of EMT including the loss of apical-basal polarity, disruption of cell-to-cell contacts (including adherent junctions, tight junctions, and desmosomes), cytoskeleton structure and ECM degradation by expressing matrix metalloproteinases. Consequently, cells ongoing EMT acquire a spindle-shape mesenchymal morphology which allows them motility and ability to degrade and invade their basal ECM [33].

## 3. EMT in PDAC

### 3.1. Activation of EMT in PDAC

**Figure 2:**
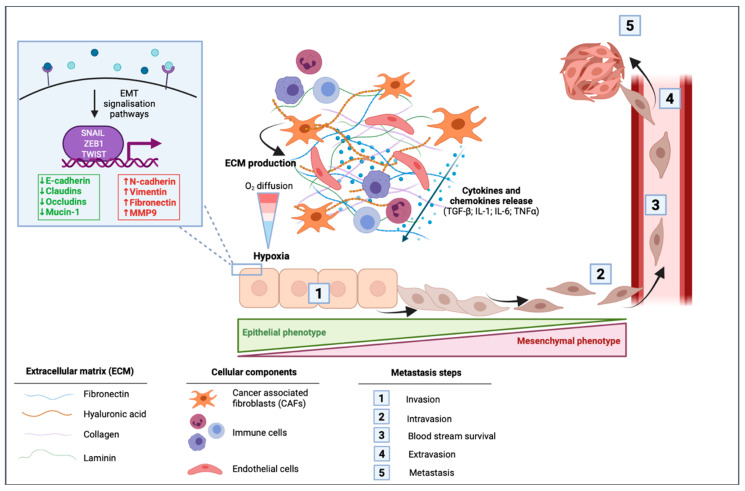
Impact of the tumor microenvironment of pancreatic ductal adenocarcinoma (PDAC) in the activation of epithelial to mesenchymal transition (EMT). Cancer-associated fibroblasts (CAFs) induce the production of extracellular matrix (ECM) that is composed of fibronectin, hyaluronic acid, collagen, and laminin. This dense desmoplastic stroma limits the diffusion of oxygen in the tumor and leads to hypoxia. CAFs release different cytokines and chemokines including the transforming growth factor β (TGF-β), interleukin 1 (IL-1), interleukin 6 (IL-6), and the tumor necrosis factor α (TNF-α). These extracellular mediators can activate signaling pathways leading to the activation of EMT in which cells switch from their epithelial phenotype to a mesenchymal phenotype with a spindle-shape morphology. EMT is then followed by intravasion of mesenchymal cells, blood stream survival, extravasion, and finally by the formation of metastasis.

In cancer cells, EMT can be activated through different stimuli. Marcucci et al. have identified five main classes of stimuli: mechanical stress, low pH and hypoxia, innate and adaptative immune responses, altered ECM and treatment with anti-tumor drugs [34]. PDAC is well known for its desmoplastic stroma, which is composed of a dense acellular extracellular matrix (ECM) infiltrated by heterogenous populations of immune, endothelial cells, and cancer associated fibroblasts (CAFs) [35]. A dense stroma can predispose the tumor microenvironment to limit delivery and diffusion of oxygen creating a hypoxic environment. The dense desmoplastic stroma composes a large part of the ECM and includes collagen, fibronectin, laminin, and hyaluronic acid. These ECM proteins are mainly produced by CAFs and in smaller amounts by cancer cells. They first have the ability to form a physical barrier, although they also can have signaling functions in EMT [36]. CAFs are able to produce various cytokines and chemokines such as TGF-β; interleukin 1 (IL-1); interleukin 6 (IL-6) and tumor necrosis factor α (TNFα), to the latter activating signaling pathways of EMT [36]. In PDAC cells, microenvironmental changes including hypoxia or TGF-β stimulation led to changes in EMT-markers within a decrease in E-cadherin and an increase in vimentin protein and mRNA levels [37] (Figure 2).

Senescence and cancer are well known to be linked. In PDAC, senescence seems to occur in earliest stages and provides tumor suppressor effects. However, several evidences indicates that senescent cells in microenvironment can have a pro tumorigenic role in part with the senescence-associated secretory phenotype (SASP) [38]. Exposure to SASP can induce cell plasticity through the stimulation of cancer cell proliferation, motility and the generation of inflammatory environment [39]. Therefore, in PDAC microenvironment SASP could participates to enhance EMT.

EMT can also be favored by mutations, the major mutation found in PDAC in more than 90% of case, is the activation of the *KRAS* oncogene [40]. *KRAS* activation can modulate the tumor microenvironment and maintaining an active stroma through the production of IL-6 and sonic hedgehog, which play an important role in the EMT process [41]. Loss of *SMAD4* is one of the fourth most common mutations found in PDAC, with an inactivation found in 60% of case, resulting in alterations in the TGF-β signaling pathway which is itself altered in 47% of PDAC cases [42]. Considering of the potential role of TGF-β in the induction of EMT, this alteration may have an impact during PDAC progression.

### 3.2. Role of EMT in PDAC Metastasis

EMT is traditionally considered as a binary phenomenon, allowing the transition from epithelial to mesenchymal phenotype, which is called “complete EMT” (cEMT). However, increased recent evidence suggests that cells undergoing EMT are heterogeneous and can express both epithelial and mesenchymal markers in a hybrid state called “partial EMT” (pEMT) [9,43,44]. Using a spontaneously metastatic genetically engineered mouse model (GEMMs) of PDAC, Aiello et al. found that EMT subtypes influence the mode of cell migration in metastasis. Tumor cells in cEMT subtype lacking E-cadherin protein preferentially disseminate as single cells. On the other hand, in the pEMT subtype, tumor cells expressing both epithelial and mesenchymal markers preferentially disseminate through collective migration [9]. EMT is a key step in metastasis which is a late stage of tumorigenesis. However, in pancreatic carcinomas EMT has been identified to occur at early stage. Rhim et al., assume that EMT occurs at preinvasive stages leading the seeding to distant organs to occur before and simultaneously to the primary tumor formation [10].

Despite clear evidences of EMT implication in tumor metastasis, the exact functions of EMT in cancer are still debated. Indeed, a study has challenged the role of EMT in metastasis, precisely on effects of EMT-TF SNAIL and TWIST in pancreatic cancer. Zheng et al. used the PDAC model KPC (Pdx1-cre; KRAS^G12D^; p53^R172H^) in mouse in which they independently knockout TWIST or SNAIL. Despite inducing suppression of EMT, the loss of SNAIL or TWIST did not alter cancer progression or local invasion or metastasis. The authors therefore claim that EMT is dispensable for metastasis. In this study, EMT-TF knockout was also correlated with chemosensitivity to gemcitabine, which has been attributed to the increased expression of nucleotide transporters, authors conclude therefore EMT induces chemoresistance in pancreatic cancer [45]. Similar observations have been made in breast cancer for lung metastasis [46]. Krebs et al. used the same KPC mouse model with targeting another key EMT-TF ZEB1. In contrast to depletion in TWIST or SNAIL, ZEB1 knockout has impair multiple stages of tumorigenesis including precursor lesion formation, tumor grading, invasion, and metastasis that clearly demonstrate a key role of ZEB1 in the in vivo tumor progression of pancreatic cancer from premalignant lesion towards metastasis [47]. Previously another study using a short hairpin knockdown of ZEB1 in mice has also highlighted the importance of ZEB1 in tumor cells dissemination and in the tumor cells capacity to initiate tumor in pancreatic cancer [48]. Putting together these studies shows a trend to functional differences of EMT-TFs, although SNAIL and TWIST seem to be dispensable ZEB1 is on the contrary a key factor that appears to be not compensable by another EMT-TF.

## 4. Clinical Implication of EMT in PDAC

### 4.1. EMT in Prognosis of Patients with PDAC

Different PDAC molecular subtypes have been identified. Among them, Collisson et al. have proposed the quasi-mesenchymal (QM), classical, and exocrine like subtypes [49]; Moffitt et al. proposed the basal-like and the classical subtypes [50]; and Bailey et al. proposed four different subtypes: squamous, pancreatic progenitor, immunogenic and aberrantly differentiated endocrine exocrine (ADEX) [51]. Most studies are according to define two accepted subtypes of pancreatic cancer: classical, that can be associated to an epithelial phenotype, and basal-like, that can be associated to a mesenchymal phenotype. A recent study performed a meta-analysis including five independent cohorts with patients with PDAC in order to assess the overall survival according to these different subtypes. Authors found that patients with the Moffitt basal-like subtype have significantly worse prognosis compared to the Moffit classical subtype (HR = 1.98, *p* < 0.0001) [52]. Similar observations have been described with a worse prognosis for the Collisson QM subtypes and the Bailey squamous subtypes which are included in the accepted basal-like subtype.

Moreover, other observations have been made in pancreatic carcinomas, cancer cells exhibiting EMT being associated with poor survival in patients [53], and ZEB1 expression is correlated with aggressive precursor lesions and with poor outcomes [47]. A study has performed an analysis of single-cell transcriptome of patients with PDAC. The authors defined a cell population with EMT characteristics and found that high number of EMT tumor cells was significantly associated with shorter patient survival (HR = 2.76, *p* < 0.0001) [54].

Analysis of circulating tumor cells (CTC), that is, cells shed from the primary tumor or its metastases that migrate into the blood stream, represents a promising noninvasive approach to predict tumor recurrence and prognosis. The process of EMT is found in CTC, and there are three different subpopulations: epithelial (E-CTC); epithelial/mesenchymal (E/M-CTC) and mesenchymal (M-CTC). In a cohort of 107 patients with PDAC, a study has identified M-CTC in 45.8% of patients. The presence of M-CTC was positively correlated with TNM stage (*p* < 0.01) and distant metastases (*p* < 0.01) [55]. Another recent study has analyzed CTC at two different sites, first at the portal vein which is the initial access site to CTCs and at peripheral vein in 39 patients prior to a surgical resection combined with an adjuvant therapy. They found a spatial heterogeneity of CTC with a significantly lower percentage of M-CTC in peripheral blood than in portal blood. Furthermore, the authors found a correlation between high M-CTC level in portal blood and significant decreased recurrence-free survival in patients after resection (HR = 8.576; *p* < 0.0001) [56].

There is an ongoing clinical trial detecting and measuring mRNA levels of genes involved in EMT in peripheral blood samples of tumor in patients with pancreatic cancer to determine the presence of cancer, the progression and risk of recurrence (NCT04323917). The study will provide a molecular profile of EMT-TFs variations in blood with early, intermediate, or advanced pancreatic cancer with respect to disease progression and administered treatments. The main aim of the study is firstly to assess the use of EMT in diagnosis and secondly to identify biomarkers suitable to select patients likely to respond to medical and surgical treatments.

Taking together all these studies clearly indicate a worse prognosis for patient with a mesenchymal phenotype. A characterization of patient EMT subtype might be a potential interesting biomarker for PDAC prognosis and in predicting tumor recurrence.

### 4.2. EMT and Chemoresistance

In the management of PDAC the standard of care was for many years gemcitabine, after demonstrating a significant improvement in overall survival versus fluorouracil-based regimen [57]. The FOLFIRINOX protocol (combination of fluorouracil, leucovorin, irinotecan, and oxaliplatin) is now the first-line option in adjuvant settings or for patients with metastatic disease [58,59]. Nab-paclitaxel represents also an alternative in association with gemcitabine in patients with metastatic disease [60]. Recently, the use of Olaparib, a poly(adenosine diphosphate-ribose) polymerase (PARP) inhibitor, in patients with metastatic disease and with germline *BRCA1/2* mutation has been approved [61]. Despite different therapeutic options, the efficiency of the latter is not optimal and treatment resistance is still a major problem in PDAC management.

Plasticity given by EMT in tumor cells leads to tumor heterogeneity, making drugs less specific and treatments less efficient [15]. EMT process has been reported to confer multidrug resistance in human PDAC cell lines especially gemcitabine and 5-fluorouracil (5-FU) [62]. In their study Arumugam et al. have silenced ZEB-1 in different PDAC cell lines, reversing therefore EMT. They observed significant increase of apoptotic cell death after gemcitabine, fluorouracil, and cisplatin treatment in drug-resistant cell lines. However, they also studied the effect of silencing SNAI1, SNAI2, and TWIST but did not observed either correlation with drug sensitivity [62]. Another in vitro study using two cell lines resistant to gemcitabine has showed that gemcitabine-resistant pancreatic tumor cells are associated with morphologic and molecular alteration typically associated with EMT, an invasive and aggressive phenotype [63]. In a subsequent study, the same team has identified the notch pathway as being at the origin of the EMT in gemcitabine-resistant pancreatic cells [64]. In vivo models of PDAC also demonstrated chemoresistance to gemcitabine in EMT cells, and on the other hand, an enhanced sensitivity for EMT-suppressed cancer cells to gemcitabine [45].

Patients with a basal-like subtype have a worse prognosis, and basal-like tumors also have a poor response to chemotherapy. O’Kane et al., in a cohort of 195 patients with pancreatic cancer, found an overall response rate of 33% in classical PDAC versus 10% in basal-like PDAC (*p* = 0.02). In basal-like phenotype patients treated by modified FOLFIRINOX the progression rate was much higher with 60% vs. 15% in classical PDAC (*p* = 0.0002) [65].

### 4.3. EMT as a Biomarker in the Choice of Treatment and to Predict Chemoresistance

Useful biomarkers are still needed for the diagnosis, prognosis, and the management of adjuvant therapies in patients with PDAC. Within the large tumoral heterogeneity, GATA6 has been identified to be recurrently amplified in the genome of pancreatic lineage with a frequency of copy number gains in GATA6 higher in classical than in basal-like subtype (*p* = 0.0015) [66]. GATA6 belongs to a family of transcription factors that can activate or repress gene expression. It has an important role in cell differentiation in pancreatic lineage [67]. Furthermore, GATA6 has pro-epithelial and anti-EMT function. It can have a direct action through the regulation of epithelial and mesenchymal genes and an indirect action through the regulation of pro-epithelial and pro-mesenchymal transcription factors [68]. Martinelli et al. have studied the effect of GATA6 expression in patients with PDAC: High expression of GATA6 was significantly associated with longer overall survival, compared to GATA6 low expression (13.1 months versus 4.6 months respectively, (*p* = 0.003). Authors also showed that patients with GATA6 low expression tumors do not benefit from adjuvant 5-FU/leucovorin with a lower survival than patients with GATA6 high tumors (*p* = 0.018). By contrast GATA6 expression was not associated with the survival of patient receiving adjuvant gemcitabine [68].

O’Kane et al. have demonstrated that basal-like PDAC can be identified by GATA6 expression by RNA-Seq and in situ hybridization. In their study, GATA6 expression appears as prognostic factor (*p* = 0.02) [65]. Altogether these results highlight GATA6 as a potential predictive biomarker in risk stratification, prognostic and predictive of response to therapy.

A recent study established an EMT-related genes signature which can predict disease free survival (DFS) and adjuvant chemotherapy response. Authors identified an 8-gene signature (DLX2, FGF9, IL6R, ITGB6, MYC, LGR5, S100A2, and TNFSF12) which could be used in order to calculate a risk score. They validated this signature in two large public cohorts and found patients in high-risk group, who had a significant decreased DFS time than patients in low-risk group. Furthermore, patients with a low-risk score have higher response rates to adjuvant chemotherapy compared to patients with high-risk score (61% vs. 32%, *p* < 0.001). Among patients who received gemcitabine monotherapy, patients with low-risk score had a significantly longer DFS (*p* = 0.0022). However for patients who received FOLFIRINOX chemotherapy, the low-risk group had longer DFS although this difference was not statistically significant (*p* = 0.094) [69]. This signature highlights the potential use of EMT biomarkers as a novel model to predict response to chemotherapy.

## 5. EMT as a New Target?

EMT plays a pivotal role in tumor formation, progression, metastasis, and in chemotherapeutic resistance highlighting its association with the poor prognosis of PDAC patients. Targeting EMT might be considered through different strategies, however signaling pathways implied EMT are also linked with others cellular mechanisms which could complicate the targeting approach with direct and indirect effects on cancer cells.

### 5.1. Inhibition of Extracellular Mediators and Their Receptors

**Figure 3:**
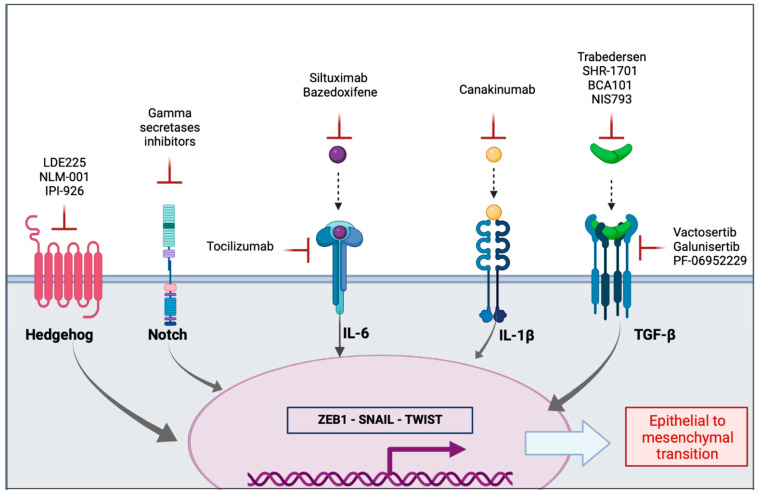
Agents in clinical trials targeting ligands or receptors of signaling pathways implied in the activation of epithelial to mesenchymal transition in pancreatic cancer.

There is a multiplicity of extracellular mediators and signaling pathways involved in the induction of EMT process. Extracellular mediators are mostly cytokines, chemokines, or growth factors and can be targeted through antagonists, monoclonal antibody (mAb) or through inhibitors of receptor tyrosine kinases (RTK) (Figure 3 and Table 1).

#### 5.1.1. Transforming Growth Factor β

One of the most potent inducers of EMT in PDAC is TGF-β, a multifunctional cytokine belonging to the transforming growth factor superfamily. Different kind of inhibitors have been designed to target the TGF-β pathway, among them inhibitor of RTK Vactosertib (TEW-7197, MedPacto) is an orally bioavailable inhibitor of the serine/threonine kinase TGF-β receptor I (TGF-βRI). There is currently a multicenter, open-label phase Ib clinical trial to evaluate the safety, tolerability, and efficacy of vactosertib in combination with FOLFOX in patients with metastatic PDAC who have failed in first-line gemcitabine and Nab-paclitaxel (NCT03666832). Another study will evaluate the safety and the recommended dose of vactosertib in combination with nanoliposomal irinotecan with 5-FU and leucovorin (NaI-IRI/FL) in patients with metastatic PDAC who have failed first-line gemcitabine and nab-paclitaxel (NCT04258072).

Galunisertib (LY21557299, Eli Lilly &Co) is a TGF-βRI kinase inhibitor. It has been evaluated in phase II study in advanced or metastatic unresectable pancreatic cancer in combination with gemcitabine (NCT01373164). This clinical trial demonstrated an improvement of an overall survival with 8.9 versus 7.1 month for galunisertib and placebo group, respectively [70]. There are two others clinical trials which studied galunisertib in metastatic pancreatic cancer, one of them in combination with gemcitabine (NCT02154646). In this phase I clinical trial, the authors found an acceptable safety and tolerability profile with evidence of efficacy in patients with advanced or metastatic pancreatic cancer [71]. In the second phase I clinical trial, galunisertib was in combination with the anti-PD-L1 Durvalumab (NCT02734160). The authors established recommended doses for both drugs however, they found a limited clinical activity in this patients population [70].

Others small molecules inhibitors of TGF-β receptor kinase activity LY2109761, LY580276, SD-093 has demonstrated promising effects with acting on EMT and tumor cell migration in preclinical models of pancreatic cancers [72].

Another well-represented class of therapeutic approach is antibodies targeting TGF-β. The anti-TGF-β mAb NIS793 (Novartis Pharmaceuticals) targets directly TGF-β ligands and prevents therefore the activation of associated signaling pathways. It is currently in phase I clinical trial in patients with advanced malignancies, including pancreatic cancer, in combination with the anti-PD1 spartalizumab (NCT02947165) and in phase II clinical trial in first line of metastatic PDAC with and without spartalizumab in combination with gemcitabine and nab-paclitaxel (NCT04390763).

BCA101 (Bicara Therapeutics) is an EGFR/TGF-β fusion mAb; it is studied in phase I alone or in combination with the anti-PD1 pembrolizumab in patients with EGFR-driven advanced solid tumors including pancreas cancer with *KRAS* G12D mutation (NCT04429542). The anti-PD-L1/TGF-βRII fusion protein SHR 1701 (Jiangsu HengRui Medicine Co) is composed of an anti-PD-L1 mAb bounding via the Fc region the N-terminal extracellular domain of TGF-βRII. This compound is currently under phase Ib/II in combination with gemcitabine and albumin paclitaxel in first-line treatment of advanced and metastatic pancreatic cancer (NCT04624217).

There are other agents targeting TGF-β signaling pathway, the antisense oligonucleotides Trabedersen (AP12009, Antisense Pharma GmbH/Isarna) targeting TGF-β2 expression has been studied in pancreatic cancer in a phase I clinical trial to evaluate safety and tolerability (NCT00844064). PF-06952229 (Pfizer) is an orally bioavailable inhibitor of TGF-βRI. It is in phase I in previously treated patients with advanced or metastatic cancers, including pancreatic neoplasm that may have high TGF-β signatures and EMT expression (NCT03685591).

#### 5.1.2. Interleukins

IL-6 is another multifunctional cytokine that is highly present through the secretion by CAFs in the stroma of PDAC [73] and is related to EMT process [74].

Tocilizumab, a recombinant mAb, is directed against the IL-6 receptor (IL-6R) and therefore blocks the binding of IL-6 to its receptor and the signaling activation. There is currently a phase II clinical trial exploring the synergy between Tocilizumab, Ipilimumab (mAb anti CTLA-4), Nivolumab (mAb anti-PD-1) and radiations in patients with locally advanced or metastatic pancreatic tumors who have progressed after a first line of systemic chemotherapy (NCT04258150). Another phase II is currently recruiting patients with unresectable pancreatic carcinoma to evaluate whether tocilizumab with gemcitabine and Nab-paclitaxel is more effective than gemcitabine and Nab-paclitaxel alone (NCT02767557). A phase IB/II, open-label, multicenter, randomized study, is recruiting patients with metastatic PDAC to study the efficacy and safety of multiple immunotherapy-based treatment combinations. Two cohorts will be enrolled in this study, the first will consist of patients who have received no prior systemic therapy and the second cohort will consist of patients who have received one line of prior systemic therapy. In each cohort patients will be assigned to one of several treatment arms with the following drugs: nab-paclitaxel, gemcitabine, oxaliplatine, leucovorin, fluorouracil, atezolizumab, cobimetinib, PEGPH20, BL-8040, selicrelumab, bevacizumab, RO6874281, AB928, tiragolumab, and tocilizumab (NCT03193190). Siltuximab (CNTO 328) is another mAb directly targeting the IL-6, evaluated in monotherapy in a phase I/II study in patients with solid tumors including pancreatic neoplasms. Results are still pending (NCT00841191). There is also a phase IB/II trial study with siltuximab in combination with spartalizumab in patients with metastatic pancreatic cancer (NCT04191421).

Bazedoxifene is a third-generation selective estrogen receptor modulator (SERM) and it has also been demonstrated to inhibit IL-6/glycoprotein 130 (GP130) protein–protein interactions [75]. A prospective, non-randomized trial is recruiting patients with metastatic pancreatic adenocarcinoma to measure IL-6 pathway modifications on metastasis biopsy in patients before and after treatments with bazedoxifene in addition to chemotherapy (NCT04812808). Bazedoxifene has been investigated in vitro in combination with anti-interleukin 8 (IL-8), that is overexpressed in pancreatic cancer tissues and blood circulation, and is associated to metastasis and poor prognosis as well as IL-6 [76]. In this study, two small-molecules targeting IL-8 reparaxin and SCH527123 were used in combination with bazedoxifene in PDAC and triple-negative breast cancer (TNBC) cell lines. The results showed that combination with bazedoxifene with reparaxin or SCH527123 synergistically inhibited cell migration and viability of PDAC and TNBC cells. This is consistent with previous observations in melanoma in which blockade of IL-6 and IL-8 inhibits invasiveness [77]. Combination of anti-IL-6 and anti- IL-8 may provide more effective treatment for different cancer types; however, to our knowledge, there is no ongoing clinical trial using this combination in patients with PDAC.

Interleukin 1β (IL-1β) is another cytokine who are present in the tumor microenvironment of PDAC [36]. The mAb anti-IL-1β canakinumab (ACZ885) is study in combination with spartalizumab and the chemotherapy combination of gemcitabine and nab-paclitaxel in a phase Ib in metastatic pancreatic cancer patients (NCT04581343).

#### 5.1.3. Sonic Hedgehog Signaling Pathway

The sonic hedgehog signaling pathway promotes EMT in PDAC with increasing motility and invasiveness of pancreatic cancer cells [78] Downregulation of the hedgehog pathway in combination with chemotherapy improved outcomes in animal models of PDAC [79,80]; therefore, clinical trials have been conducted in patients with PDAC. Vismodegib (GDC-0449), a hedgehog inhibitor that targets the hedgehog–ligand cell surface receptors, has been evaluated in a phase II trial in combination with gemcitabine and nab-paclitaxel in metastatic PDAC. This study reveals that adding vismodegib to conventional chemotherapy did not improve efficacy compared as chemotherapy alone [81]. Authors generate hypothesis to explain this lack of efficiency. They hypothesized that there is a chemotherapy-induced mechanism that leads to hedgehog signaling activation despite inhibitors administration. They also postulate that hedgehog inhibitors did not cause a change in stromal component, limiting therefore their action.

Other clinical trials failed to demonstrate a benefit or detriment in progression-free survival. However, there are still currently clinical trials with hedgehog inhibitors: LDE225 (NCT02358161; NCT01485744), NLM-001 (NCT04827953), and IPI-926 (NCT01130142) in advanced pancreatic cancer.

#### 5.1.4. Notch Signaling Pathway

Notch signaling pathway is involved in the acquisition of EMT phenotype. Furthermore, downregulation of notch signaling led to partial reversal EMT phenotype in vitro [64], suggesting a potential targeted approach in the management of patients with PDAC. RO4929097 is a small-molecule gamma-secretase inhibitor who blocking activation of notch receptor. It has been studied in phase I in combination with gemcitabine and in phase II in monotherapy [82,83]. However, due to the lack of clinical activity, the development of this gamma-secretase inhibitor has been discontinued. MK0752 another gamma secretase inhibitor has been studied in phase I in combination with gemcitabine in patients with stage III and IV pancreatic cancer that cannot be removed by surgery. In this study MK-0752 and gemcitabine had the same activity than with gemcitabine alone [84]. To our knowledge, there is no other clinical trial targeting the notch signaling pathway in pancreatic cancer.

Targeting EMT could be beneficial for a plethora of cancers. Furthermore, it has been demonstrated a non-specificity of tumor stroma. Activity of CAFs is not cancer-specific, indeed CAFs isolated from different types of tumors have been able to influence the phenotype of breast cancer cell line with a tumor type unspecific mechanism [85]. This non-specificity might give a great opportunity to the development of therapeutics.

### 5.2. MicroRNAs

MiRs are involved in various biological process of pancreatic cancer progression including EMT. Among the large number of miRs implied in PDAC the miRs-200 family has a major role in the regulation of EMT. It has been demonstrated that EMT-TF ZEB1 can activate EMT through the repression of miRs-200 which inhibit stemness in pancreatic cancer [48]. Expression of miR-200b, miR-200c, let-7b, let-7c, let-7d, and let-7e was downregulated in cell lines resistant to gemcitabine with EMT morphological characteristics. After a re-expression of miR-200 in these cell lines, there was a morphological reversal to epithelial phenotype [86]. Re-expression of these miRs may be a potential new strategy to reverse EMT process. However, there are a plethora of difficulties in miRs therapeutic strategy. MiRs have a high molecular weight and a negative charge which represents a first obstacle to the membrane penetration and cellular uptake. In vivo the stability of miRs is also poor, they can be rapidly degraded by nucleases in blood stream. Another important risk with delivery of miRs is an inappropriate biodistribution and the potential off-target effects [87].

A study developed a multifunctional nanoscaffold targeting miR-21, which is also implied in cancer initiation and progression of pancreatic cancer. Authors developed a combine therapy of miR-21 antisens oligonucleotide and gemcitabine using a polyethylene glycol–polyethylenimine–magnetic iron oxide nanoparticles [88]. This nanocomplex induced the suppression of EMT leading to a decrease of the proliferation, migration, and invasion of pancreatic cancer cell in vitro and leading to reduced liver metastasis in vivo.

There are many studies focusing on the improve delivery of miRs to bypass the biological barriers, nevertheless to our knowledge there is no ongoing clinical trial in pancreatic cancer.

### 5.3. Nanomedicine to Target EMT

Nanoparticles are extensively studied as drug delivery vehicles or as cancer inhibiting agents. Nanoparticles are valuable drug-delivering agents due to their small size, biocompatibilities and biodegradability properties, functionalization, and their enhanced permeability and retention (EPR) effect. Furthermore, depending on their physico-chemical and structural properties nanomaterials possess the ability to inhibit EMT [89].

Among inorganic metallic nanoparticles, gold nanoparticles have been reported to inhibit cell proliferation and metastasis and increase the sensitivity to gemcitabine in vitro and in vivo models of pancreatic cancers by reverting the mesenchymal phenotype to epithelial phenotype [90].

The use of nanomaterials as carrier of natural substances has been reported in different studies to treat cancer by suppressing EMT. Among natural substances, anthothecol and α-mangostin present interesting anticancer properties, particularly α-mangostin was found to suppress EMT by inhibiting MMP9 and MMP2 and increasing E-cadherin expression [91]. However, these substances have poor bioavailability and solubility. To overcome their poor pharmacokitenic profile, anthothecol and α-mangostin have been encapsulated into the core of poly (d,l-lactic-co-glycolic acid) (PLGA) nanoparticles (Mang-NPs and Antho-NPs). Mang-NPs andAntho-NPs were found to inhibit EMT with an inhibition of N-cadherin and EMT-TFs SNAIL and ZEB1, and an upregulation of E-cadherin in pancreatic cells [92,93]. PLGA was also used to deliver salinomycin, a monocarboxylic polyether antibiotic which has an activity against pancreatic cancer stem cells [91]. Salinomycin loaded in PLGA nanoparticles resulted in EMT inhibition with an upregulation of E-cadherin and β-catenin [94].

Despite the various advantages of the use of nanoparticles in drug delivery or as anticancer agents, nanomedicine still need improvements to overcome the potential sides effects of nanomaterial such as cytotoxicity, inducing inflammation and fibrosis, and neoplastic transformation.

## 6. Conclusions

EMT is a well-known process in both physiological and pathological states. In PDAC, EMT is strongly associated to cancer progression with cell migration and metastasis. The EMT process also confers chemotherapy resistance that partly explains the poor prognosis associated to mesenchymal phenotype in patients with pancreatic cancer. EMT status might be an interesting biomarker to determine prognosis, predict tumor recurrence, and to stratify the management of patients with pancreatic malignancy. EMT is induced by different stimuli which are abundant in the desmoplastic stroma of PDAC. Activation of EMT is regulated through complex networks by transcriptional regulators, signaling pathways, and miRs; however, the comprehension of the molecular biology of EMT in PDAC still need to be elucidated. Several extracellular mediators of EMT and their associate receptors are currently targeted in clinical trials. The clinical potential of anti-EMT compounds is encouraging and might be beneficial for patients.

## 7. Methods

### 7.1. Search Strategy

This review was conducted through a systematic review according to the directions denoted by the Preferred Reporting Items for Systematic reviews and Meta-Analysis (PRISMA). To investigate the entirety of the published studies on epithelial to mesenchymal transition in patients with pancreatic ductal adenocarcinoma, a comprehensive literature search of the electronic database PubMed was performed up to July 2021. Studies were selected using the following search terms: “PDAC” and “EMT”.

### 7.2. Figures

Figures have been created with Biorender (https://biorender.com/).

## Figures and Tables

**Table 1:** Different agents that target stimuli and signaling pathway associated with EMT in pancreatic cancer in clinical trials.

Drug Name	Combination	Functional Class	Study Population	Phase	Study NCT Registry Number
Vactosertib (TEW-7197)	FOLFOX	Inhibitor of the serine/threonine kinase TGF-βR1	Metastatic PDAC who have failed first-line gemcitabine and nab-paclitaxel	Ib	NCT03666832
	Nanoliposomal irinotecan with 5-FU and leucovorin		Metastatic PDAC	II	NCT04258072
Galunisertib (LY2157299)	Durvalumab	TGF-βR1 kinase inhibitor	Recurrent or refractory metastatic pancreatic cancer	Ib	NCT02734160
	Gemcitabine		Inoperable or metastatic pancreatic cancer	Ib	NCT02154646
	Gemcitabine		Advanced or metastatic unresectable pancreatic cancer	Ib/II	NCT01373164
Trabedersen (AP 12009)	_____	Antisense oligonucleotide specific for the mRNA TGF-β2	Advanced tumors known to overproduce TGF-β2 (Pancreatic neoplasm)	I	NCT00844064
SHR-1701	Gemcitabine and albumine paclitaxel	Bifunctional fusion protein targeting PD-L1 and TGF-β	Advanced/Metastatic pancreatic cancer in first line treatment	Ib /II	NCT04624217
PF-06952229	_____	TFG-β receptor I inhibitor	Advanced solid tumors (Pancreatic neoplasms)	I	NCT03685591
BCA101	Alone or with pembrolizumab	EGFR/TGF-β fusion mAb	Patients with EGFR-driven advanced solid tumors (Pancreas cancer with *KRAS* G12D mutation)	I	NCT04429542
NIS793	Spartalizumab	Anti-TGF-β mAb	Advanced malignancies (Pancreatic cancer)	I	NCT02947165
	With and without spartalizumab in combination with gemcitabine and nab-paclitaxel		First-line in metastatic PDAC	II	NCT04390763
Tocilizumab	Gemcitabine and nab-paclitaxel	Anti -IL-6 Receptor mAb	Unresectable pancreatic carcinoma	II	NCT02767557
	Ipilimumab, nivolumab and radiation		Advanced pancreatic cancer	II	NCT04258150
	Nab-paclitaxel, gemcitabine, oxaliplatine, leucovorin, fluorouracil, atezolizumab, cobimetinib, PEGPH20, BL-8040, selicrelumab, bevacizumab, RO6874281, AB928, tiragolumab		Metastatic PDAC	I/II	NCT03193190
Siltuximab	_____	Anti-IL-6 mAb	Solid tumors (Pancreatic neoplasms)	I/II	NCT00841191
	Spartalizumab		Metastatic pancreatic cancer	Ib/II	NCT04191421
Bazedoxifene	Gemcitabine and nab-paclitaxel	Selective estrogen receptor modulator—Inhibitor of IL-6/glycoprotein 130	Metastatic pancreatic cancer	-	NCT04812808
Canakinumab (ACZ885)	Spartalizumab, gemcitabine and nab-paclitaxel	Anti-IL-1-β mAb	Metastatic pancreatic cancer	Ib	NCT04581343
LDE225	Gemcitabine and nab-paclitaxel	Hedgehog inhibitor	Locally advanced or metastasized pancreatic cancer	I/II	NCT02358161
	Fluorouracil, leucovorin, oxaliplatin, irinotecan		Untreated advanced pancreatic cancer	Ib	NCT01485744
NLM-001	Gemcitabine and nab-paclitaxel, zalifrelimab		Advanced pancreatic cancer	Ib/IIa	NCT04827953
IPI-926	Gemcitabine		Metastatic pancreatic cancer	Ib/II	NCT01130142

## Data Availability

Data sharing not applicable.
